# HER2-signaling pathway, JNK and ERKs kinases, and cancer stem-like cells are targets of Bozepinib

**DOI:** 10.18632/oncotarget.1962

**Published:** 2014-05-13

**Authors:** Alberto Ramírez, Houria Boulaiz, Cynthia Morata-Tarifa, Macarena Perán, Gema Jiménez, Manuel Picon-Ruiz, Ahmad Agil, Olga Cruz-López, Ana Conejo-García, Joaquín M. Campos, Ana Sánchez, María A. García, Juan A. Marchal

**Affiliations:** ^1^ Department of Health Sciences, University of Jaén, Jaén, Spain; ^2^ Department of Human Anatomy and Embryology, University of Granada, Granada, Spain; ^3^ Biopathology and Medicine Regenerative Institute (IBIMER), University of Granada, Granada, Spain; ^4^ Biosanitary Institute of Granada (ibs.GRANADA), Hospitales Universitarios de Granada-Universidad de Granada, Granada, Spain; ^5^ Braman Family Breast Cancer Institute, Sylvester Comprehensive Cancer Center, University of Miami, Miller School of Medicine, Miami, FL, USA; ^6^ Department of Pharmacology and Neurosciences Institute, Faculty of Medicine, Spain; ^7^ Department of Pharmaceutical and Organic Chemistry, Faculty of Pharmacy, University of Granada, Granada, Spain; ^8^ Andalusian Public Health System Biobank, Granada, Spain; ^9^ Department of Oncology, Virgen de las Nieves, University Hospital, Granada, Spain

**Keywords:** HER-2, bozepinib, protein kinases, cancer stem-like cells, metastasis

## Abstract

Identification of novel anticancer drugs presenting more than one molecular target and efficacy against cancer stem-like cells (CSCs) subpopulations represents a therapeutic need to combat the resistance and the high risk of relapse in patients. In the present work we show how Bozepinib [(*RS*)-2,6-dichloro-9-[1-(*p*-nitrobenzenesulfonyl)-1,2,3,5-tetrahydro-4,1-benzoxazepin-3-yl]-9*H*-purine], a small anti-tumor compound, demonstrated selectivity on cancer cells and showed an inhibitory effect over kinases involved in carcinogenesis, proliferation and angiogenesis. The cytotoxic effects of Bozepinib were observed in both breast and colon cancer cells expressing different receptor patterns. Bozepinib inhibited HER-2 signaling pathway and JNK and ERKs kinases. In addition, Bozepinib has an inhibitory effect on AKT and VEGF together with anti-angiogenic and anti-migratory activities. Moreover, the modulation of pathways involved in tumorigenesis by Bozepinib was also evident in microarrays analysis. Interestingly, Bozepinib inhibited both mamo- and colono-spheres formation and eliminated ALDH+ CSCs subpopulations at a low micromolar range similar to Salinomycin. Bozepinib induced the down-regulation of c-MYC, β-CATENIN and SOX2 proteins and the up-regulation of the GLI-3 hedgehog-signaling repressor. Finally, Bozepinib shows in vivo anti-tumor and anti-metastatic efficacy in xenotransplanted nude mice without presenting sub-acute toxicity. These findings support further studies on the therapeutic potential of Bozepinib in cancer patients.

## INTRODUCTION

Cancer represents the second cause of death by disease in the world, being colorectal and breast cancer two diseases with important incidence in most countries [1]. Some of the main signaling pathways involved in cancer are related with cell proliferation, differentiation and survival, which represent exciting targets for developing new anti-tumor drugs. In this way, the epidermal growth factor receptors EGFR and HER2 are frequently over-expressed in several human cancers of epithelial origin and play essential roles in the development and progression of cancer [2, 3]. HER2 is over-expressed in several tumors, including breast and colorectal cancers [4]. In normal cells, the HER proteins regulate cell growth, survival, adhesion, migration and differentiation through a network of signaling pathways. The over-expression of this receptor in breast cancer is often associated with increased disease recurrence and a worse prognosis. Moreover, in patients where hormonal therapy is not effective, HER2 becomes a critical target for treatment [5]. Since signal transduction networks integrate multiple upstream inputs, targeting pathways downstream of the receptors could conceivably result in greater therapeutic efficacy and broader applicability. Moreover, kinase inhibitors that reduce the excessive proliferation signaling are revealed as one of the most important emergent therapies against cancer [6]. For this reason, kinome profiling has arisen as an important tool to develop targeted therapies against kinases with aberrant expression directly involved in cancer pathology [7].

The mortality rate of breast and colorectal cancer is high because of disease recurrence, which remains the major therapeutic barrier in these types of cancer. Although chemotherapy or radiotherapy can kill most bulky tumor cells and provide temporary remission, relapse occurs in most cases, as explained in the recently proposed cancer stem cell hypothesis [8, 9]. This hypothesis implies that a subset of tumor cells has the ability to self-renew and is the source of tumor initiation, progression and recurrence. These cancer stem-like cells (CSCs) may also contribute to tumor formation, metastasis, and treatment resistance. It has been found that multiple stem cell-related molecules and enzymatic activities are highly expressed in breast and colon CSCs, including c-MYC [10, 11], β-CATENIN [12, 13], SOX-2 [14, 15], ALDH1 activity [16], among others. Targeting pathways or molecules up-regulated in differentiated cancer cells and/or in CSCs populations but not in normal cells can provide new strategies for selective therapy.

Recently, our group has reported the synthesis and anticancer activities of new (*RS*)-4,1-benzoxazepin-purines against human breast and colon cancer cell lines [17]. The most active compound (*RS*)-2,6-dichloro-9-[1-(*p*-nitrobenzenesulfonyl)-1,2,3,5-tetrahydro-4,1-benzoxazepin-3-yl]-9*H*-purine, named as Bozepinib, presents very low inhibitory concentration 50 (IC_50_) values in both breast and colon cancer cells, being the most potent structure that we have reported [17, 18]. Bozepinib induces the cell death of cancer cells by apoptosis also triggering other interesting anti-tumor activities such as autophagy and senescence processes in cancer cells without inducing acute toxicity in mice [17, 18]. These effects make Bozepinib a very attractive anti-tumor agent, opening a new strategy in cancer chemotherapy with future clinical application in cancer. The aim of this work was to characterize the mechanism of action of this drug analyzing its effect over kinases, receptors and signaling pathways that are targets for new promising therapies. In addition, we evaluated its effect on enriched subpopulations of CSCs and in related proteins of CSCs pathways. Finally, we studied the *in vivo* anti-tumor activity of Bozepinib using xenograft transplants of breast and colon cancer.

## RESULTS

### Bozepinib has an antiproliferative and selective effect on breast and colon cancer cell lines

We have previously described the anti-tumor effect of Bozepinib in some breast and colon cancer cell lines [17, 18]. To test the selective action of Bozepinib against breast and colon cancer cells we determined the therapeutic index (TI). As shown in Table 1, the IC_50_ value of CCD-18Co, a non-tumor colon cell line, was almost double that in T84 and HT29 colon tumor cells and more than triple for Caco-2 and SW-480 colon cancer cell lines, showing a TI close to 9 for the SW-480 cell line. Better results were obtained in breast cancer cell lines where the IC_50_ values determined for the MCF-10A non-tumor cell line were more than five times higher that the IC_50_ determined for MCF-7 and SKBR-3 breast cancer cell lines and almost double that of the value determined for MDA-MB 468 cancer cells, presenting the MDA-MB 231 a TI equal to 11 (Table 1). These values confirm the potent anti-tumor effects of the drug and its selective cancer activity.

**Table 1:** Bozepinib shows broad anti-proliferative effects and significantly improved the therapeutic index (TI)

Cell lines	Tissue	Bozepinib	
		IC_50_ (μM)	TI
MCF-7^a^	Breast cancer	0,355 ± 0,011	5,14
MDA-MB 231^a^	Breast cancer	0,166 ± 0,063	11
MDA-MB 468	Breast cancer	0,850 ± 0,003	2,14
SKBR-3	Breast cancer	0,330 ± 0,003	5,53
MCF-10A^a^	Normal breast	1,825 ± 0,003	ND^b^
Caco-2	Colon cancer	0,631 ± 0,008	3,18
T-84	Colon cancer	1,019 ± 0,281	1,97
HT-29	Colon cancer	1,352 ± 0,281	1,48
SW-480	Colon cancer	0,235 ± 0,011	8,56
HCT-116	Colon cancer	0,570 ± 0,020	3,52
CCD-18Co	Normal colon	2,012 ± 0,012	ND^b^

IC_50_ was determined based on sulforhodamine-B assay. The data are means ± SEM of three independent experiment.

a Taken from Ref. [17].

b ND=Not determined

### HER2 signaling pathway and several proliferative kinases are inhibited by Bozepinib

**Figure 1:**
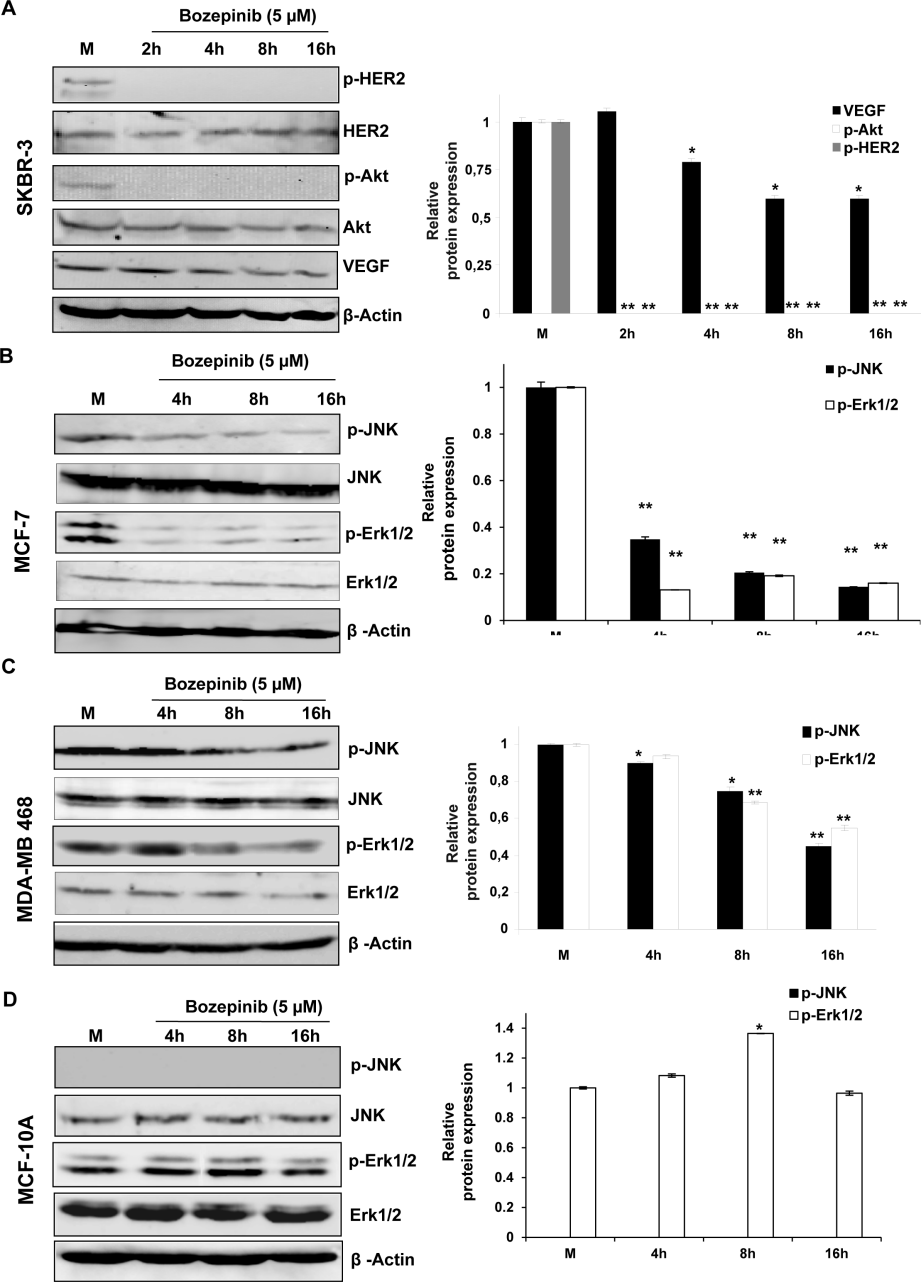
Western blot and densitometric analysis of different proteins related with cancer cell proliferation after treatment with 5 μM of Bozepinib p-HER2, HER-2, p-AKT, AKT, VEGF, p-JNK and p-ERK1/2 were analyzed after 2, 4, 8 and 16 h post-treatment in breast cancer cell lines SKBR-3 (A), MCF-7 (B) MDA-MB 468 (C) and the normal mammary epithelial cell line MCF-10A (D) and their respective mock-treated cells. β-ACTIN was used as housekeeping protein. Western blot quantification was normalized with β-ACTIN signal and relative to mock-treated cells (value 1). Data were obtained from three independent experiments performed in duplicate and are expressed as mean ± SD (**P < 0.01 vs control; *P < 0.05 vs control).

To study the effect of Bozepinib on cancer proliferation pathways, we carried out an *ex vivo* multikinase screening assay (n=36) using 5μM and 50μM (Supplementary Table S3). Bozepinib treatment at 50μM showed a significant inhibitory effect over numerous kinases such as JNK and ERKs, inhibiting also the EGFR and HER2 cellular signaling pathways. In fact, HER2, AKT2 and VEGF receptors were considerably inhibited in the screening (Supplementary Table S3). In order to analyze whether Bozepinib inhibits the HER2 signaling pathways in breast cancer cells, we treated the HER2 positive SKBR-3 cell line with 5 μM of Bozepinib and we analyzed the expression and activation of proteins involved in HER2 signaling at different times post-treatment by western blot analysis (Fig. 1A). Whereas the total level of HER2 receptor remained stable during treatment, the phophorylated form was completely inhibited after 2 hours post-treatment. Consequently, p-AKT was also inhibited and accompanied with a significant decrease in the total level of VEGF (Fig. 1A). Moreover, we also detected both the inhibition of ERKs and JNK phosphorylation in MCF-7 and MDA-MB 468 breast cancer cell lines, that was more notable in MCF-7 cells at 4 hours post-treatment and in MDA-MB 468 cell line after 8 hours post-treatment (Fig. 1B and 1C). Whereas phosphorylation of JNK was not detectable in normal MCF-10A mammary epithelial cells as previously described [19], the phosporylation of ERKs was weakly up-regulated at 8 hours post-treatment and was similar to the control non-treated cells at 16 hours post-treatment (Fig. 1D).

### Bozepinib has antiangiogenic properties and inhibits cell migration

**Figure 2:**
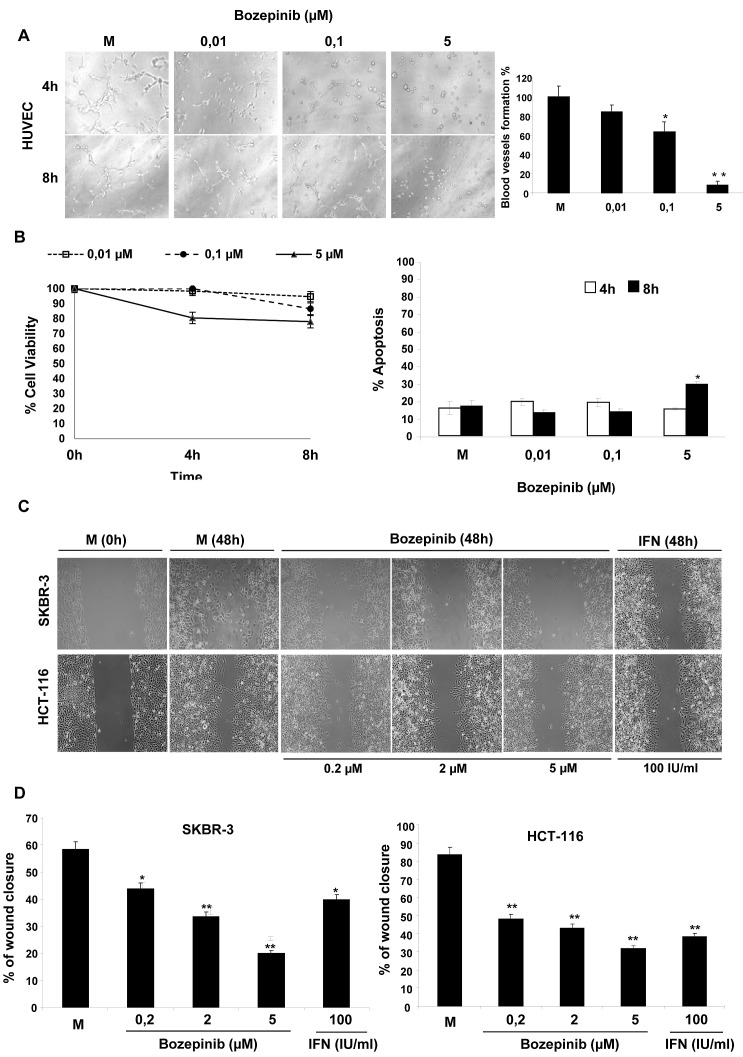
Capillary network formation and cell migration assays (A) Representative light microscopy analysis of cells at different culture stages and HUVEC grown on Matrigel™ coated wells with EGM-2 medium. Pictures were taken at 4 and 8 hours after 0 μM (Mock), 0,01 μM, 0,1 μM and 5 μM treatment with Bozepinib. Pictures from one representative experiment of three independent experiments are shown. Scale bar = 2.00 μm (left panel). Semi-quantification of the tube formation index. Bars correspond to the percentage of the number of capillary-like structures compared with the controls (HUVEC), measured after 24 hours of culture on Matrigel™. All data from three independent experiments performed in duplicate are expressed as mean ± SD (** *P* < 0.05 vs HUVEC) (right panel). (B) Cell viability (left panel) and apoptosis (right panel) of HUVEC cells were measured at 4 and 8 hours after 0 μM (Mock), 0,01 μM, 0,1 μM and 5 μM treatment with Bozepinib. (C) Wound-healing assays were performed at 0 and 48 h in SKBR-3 and HCT-116 cells in mock-treated cells (0 μM), cells treated with 0,2 μM, 2 μM and 5 μM of Bozepinib. Pictures were taken with a phase-contrast microscope (10×). 100 IU/ml of Interferon alpha (IFN) was used as an antiproliferative control. (D) The wound closure was illustrated by showing the area covered by cells 48 hours after wounding. The panel represents the quantification of the wound closure area calculated by measuring the diminution of the wound bed surface upon time using Image J software 1.47v (http://imagej.nih.gov/ij) in SKBR-3 and HCT-116 cells.

The ability of Bozepinib to suppress capillary-like structures was assessed by culturing HUVEC endothelial cells on MATRIGEL™-coated wells. As shown in Fig. 2A, HUVEC were able to form capillary-like structures. However, Bozepinib was enough to inhibit the development of these capillary-like structures in a dose-dependent manner after 4 and 8h of treatment (Fig. 2A). As shown in Fig. 2B, the HUVECs viability was maintained throughout 4 and 8 hours of treatment with low doses of Bozepinib (0,01 μM and 0,1 μM). At 4 and 8 hours the treatment with 5μM of Bozepinib presented a percentage of viability around 80% whereas the induction of apoptosis was detected only at 8 hours post-treatment showing an increase of just 15% compared to control cells. However, the vessel-like structures formation was inhibited after Bozepinib treatment at these doses (Fig. 2A).

In order to evaluate the effects of Bozepinib on cell migration, we performed a wound healing assay on SKBR-3 breast and HCT-116 colon cancer cell lines. Data showed a significant dose-dependent cell migration inhibition after 48h of treatment with 0.2, 2 and 5 μM of Bozepinib compared to mock-treated cells (Fig. 2C and 2D). The known Interferon alpha (IFN) anti-proliferative agent has been included as a control [20].

### Bozepinib treatment induces genome-wide gene expression changes in breast cancer cells

**Figure 3:**
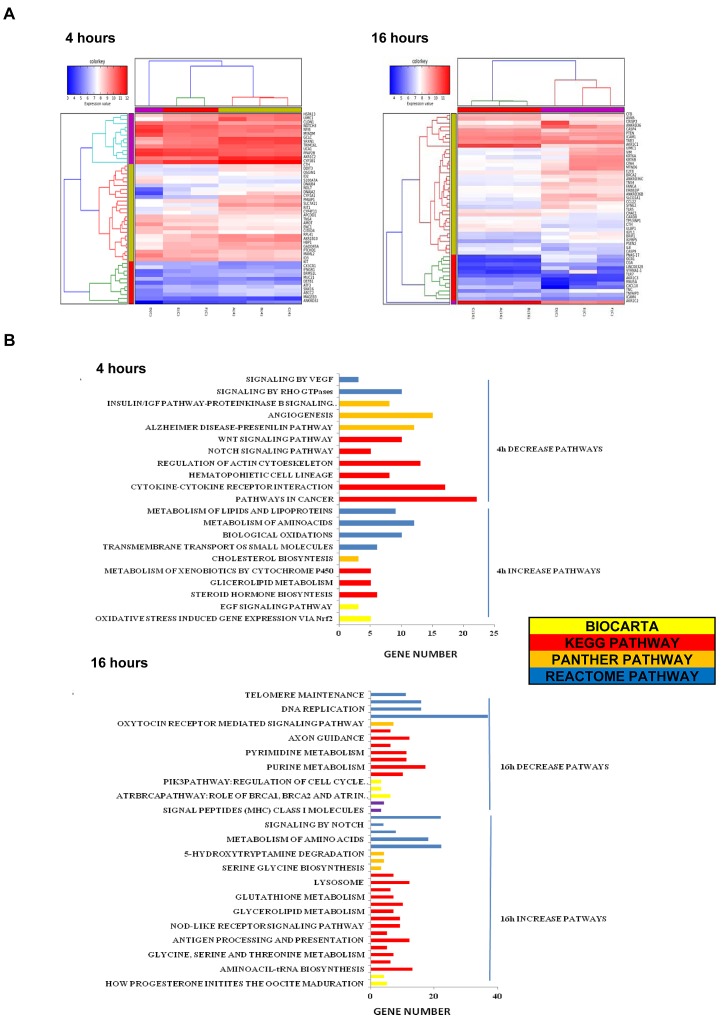
Gene expression profiling of MDA-MB 468 Bozepinib-treated breast cancer cells (A) Heat map of the 50 top fold change gene up and down-regulated at 4 and 16 hours. (B) Gene clusters pathways affected by Bozepinib-treatment after 4 hours and 16 hours. Microarrays data were processed by DAVID, KEGG, BIOCARTA, PANTHER and REACTOME.

To understand the molecular changes underlying Bozepinib exposure we performed a microarray assay comparing the effect of 5 μM in MDA-MB 468 cells at 4 and 16h post-treatment. After 4h of treatment, from 178 genes differentially expressed (cut-off values >1.5 fold change and p<0.05), 115 genes were up-regulated whereas 64 genes were down-regulated. At 16h, about 471 genes were differentially expressed, with 143 up-regulated and 328 down-regulated (Fig. 3 and Supplementary Tables S4-7). We observed the down-regulation of genes involved in breast cancer tumorigenesis (*BRCA1, DKK1, CLAUDIN1*), tumor cell progression (*NOTCH3, MAML2, VIMENTIN*), multidrug resistance (*VTRNA1-1*), cell cycle progression (*E2F8A*), DNA reparation (*BRCA1*), metastasis (*VIMENTIN, BRMS1L*) and the up-regulation of genes involved in the inhibition of angiogenesis (*CXCL10)*, apoptosis (*CASP4),* repressor of metastasis *(BRMS1L)* or genes involved in cell proliferation inhibition *(CSE),* among others (Fig. 3A).

**Figure 4:**
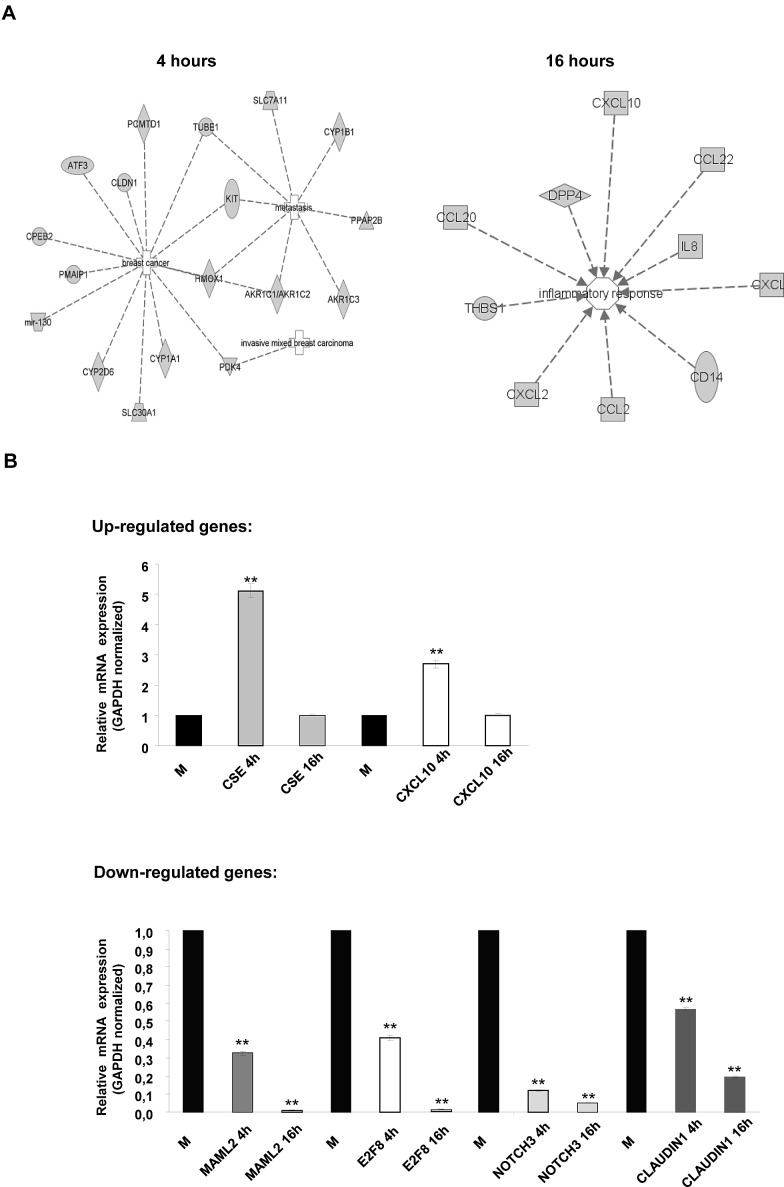
IPA pathway analysis of MDA-MB 468 cells treated with Bozepinib (A) IPA networks in MDA-MB 468 cancer cell line generated with microarray data gene. Networks were classified as breast cancer, metastasis and invasive mixed breast carcinoma at 4 hours of treatment and as inflammatory response at 16 hours of treatment. Discontinuous lines are related with indirect interaction. (B) Validation by real-time RT-PCR of up- and down-regulated related cancer genes in MDA-MB 468 after treatment with Bozepinib for 4 and 16h obtained from microarray analysis compared with mock-treated cells (value 1) and normalized with GAPDH reporter gene. Data were obtained from three independent experiments performed in duplicate and are expressed as mean ± SD (** *P* < 0.01vs control).

The gene list was then subdivided into functional categories by term enrichment score with DAVID bioinformatic analysis. A summary of functional gene clusters is shown in Fig. 3B. In addition the IPA system was used to generate networks of molecular relationships between different genes (the cut-off log ratio was set at 1.5) (Fig. 4A). We subsequently validated the microarray results in MDA-MB 468 cells using qRT-PCR. As shown in Fig. 4B, *CSE* and *CXCL10* were up-regulated while *MAML2, E2F8A, NOTCH3* and *CLAUDIN1* were down-regulated in MDA-MB 468 cells.

### Bozepinib has cytotoxicity against CSCs subpopulations and inhibits the expression of CSCs-related proteins

**Figure 5:**
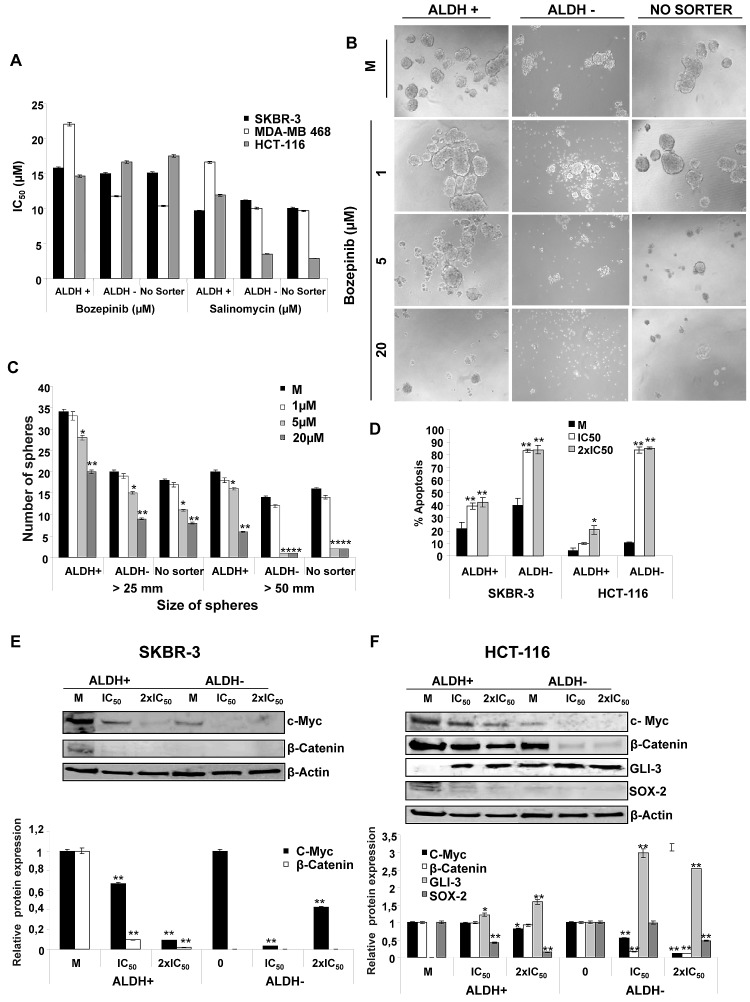
Effects of Bozepinib on ALDH subpopulations (A) Antiproliferative activities for Bozepinib and Salinomycin against subpopulations ALDH +/- and “No Sorter” cells from breast SKBR-3 and MDA-MB-468 and colon HCT-116 cell lines. (B) Anti-proliferative activity of Bozepinib on sphere formation of ALDH+, ALDH- and “No Sorter” cells from HCT-116 colon cell line at 1, 5 and 20 μM concentrations, compared with mock-treated cells. (C) Number of spheres counted in different sizes from HCT-116 ALDH+, ALDH- and “No Sorter” subpopulations. The data are from a representative experiment performed in duplicate wells. (D) Apoptosis level of ALDH+/- subpopulations from SKBR-3 and HCT-116 cell lines were determined using the Annexin V-fluorescein isothiocyanate detection kit after 24 hours of mock-treatment or treatment with the IC_50_ and twice the IC_50_ concentration of Bozepinib. All data from three independent experiments performed in duplicate are expressed as mean ± SD (**P < 0.01 vs mock-treated cells; *P < 0.05 vs mock-treated cells). (E) Western blot analysis of c-MYC and β-CATENIN in ALDH+ and ALDH- subpopulations isolated from SKBR-3 cells, and (F) GLI-3, SOX-2, c-MYC, β-CATENIN in ALDH+ and ALDH- subpopulations isolated from HCT-116 cells after mock-treatment and Bozepinib-treatment with IC_50_ and 2×IC_50_ concentration for 24 hours. Relative western blot quantification of proteins normalized with β-ACTIN signal and relative to mock-treated cells (value 1) in ALDH+ and ALDH- subpopulations. Data were obtained from three independent experiments performed in duplicate and are expressed as mean ± SD (** *P* < 0.01 vs control, *P < 0.05 vs control).

We determined the cytotoxic effect of Bozepinib on SKBR-3 and MDA-MB 468 breast and HCT-116 colon CSCs enriched subpopulations by growing them into low attachment plates with sphere forming medium for 72h. After that, CSCs were separated using ALDH activity by FACS (Supplementary Fig. S1). IC_50_ values were determined in both ALDH positive cells (called ALDH+), ALDH negative cells (called ALDH-) and in cells growing in sphere forming medium without sorter enrichment process (called No Sorter). Moreover, we used Salinomycin as reference of a potent anti-CSCs drug [21]. As shown in Fig. 5A, Bozepinib showed an IC_50_ range in SKBR-3 and MDA-MB 468 cells around that displayed by Salinomycin. Moreover, our compound was able to considerably reduce the number and size of spheres at 5 μM and to abolish the sphere-formation ability after treatment with 20 μM (Fig. 5B and 5C).

Next, we analyzed the apoptosis levels induced by Bozepinib in ALDH+/- subpopulations, showing that Bozepinib was able to induce a significant level of cell death by apoptosis in the resistant ALDH + subpopulations from both SKBR-3 and HCT-116 cell lines. Higher levels of apoptosis were detected in the ALDH- subpopulations (Fig. 5D).

Finally, we determined the modification of several described proteins involved in the stem cell phenotype of these subpopulations such as GLI-3, SOX-2, c-MYC and β-CATENIN. For this aim, both ALDH+ and ALDH- SKBR-3 breast and HCT-116 colon cancer subpopulations were treated with the IC_50_ and twice the IC_50_ of Bozepinib values for 24 hours. The c-MYC oncoprotein was detected in both SKBR-3 and HCT-116 cell lines with a high level of expression in ALDH+ subpopulations in comparison with the ALDH- subpopulations. Bozepinib induced a significant reduction of c-MYC level in ALDH+ HCT-116 cells and, interestingly, was able to inhibit its expression in ALDH+ SKBR-3-treated cells and in ALDH- subpopulations of both HCT-116 and SKBR-3 cells (Fig. 5E and 5F). The same trend was obtained for β-CATENIN expression where Bozepinib reduced its level in ALDH+ SKBR3 subpopulation. However, β-CATENIN was not expressed in ALDH- SKBR-3 cells. In addition, our compound was able to reduce β-CATENIN expression in ALDH+ HCT-116 subpopulation and to inhibit them in ALDH- HCT-116 cells (Fig. 5E and 5F).

GLI-3, a described inhibitor of stem cell properties [22] was detected in ALDH- HCT-116 cells and was absent in the ALDH+ HCT-116 subpopulations. After the Bozepinib treatment GLI-3 was strongly induced in both subpopulations. However, no changes at protein level were detected in ALDH+/- subpopulations isolated from the SKBR-3 cell line (data not shown). The stem cell transcription factor SOX2 was detected in HCT-116 ALDH+ subpopulation but was reduced after treatment with IC_50_ and practically disappeared after treatment with 2×IC_50_ of Bozepinib. In HCT-116 ALDH- subpopulation the expression of SOX2 protein was very weak (Fig. 5E and 5F).

### Bozepinib inhibits growth and metastasis of tumor xenografts in nude mice without induction of sub-acute toxicity

We have previously reported that Bozepinib has no acute toxicity in BALB/c mice even at the highest i.p. bolus dose of 200 mg/kg and p.o. bolus dose of 50 mg/kg after 2 weeks [17]. In the present work we decided to evaluate the sub-acute toxicity after 29 days of i.p. treatment with 100 mg/kg twice a week, showing that Bozepinib-treated mice presented no weight loss or unusual behavior (Supplementary Fig. S2A) and the histopathologic examination did not find any detectable toxicity in the liver or kidneys (data not shown). These data indicate that, at the concentration used, Bozepinib did not cause any systemic damage.

**Figure 6:**
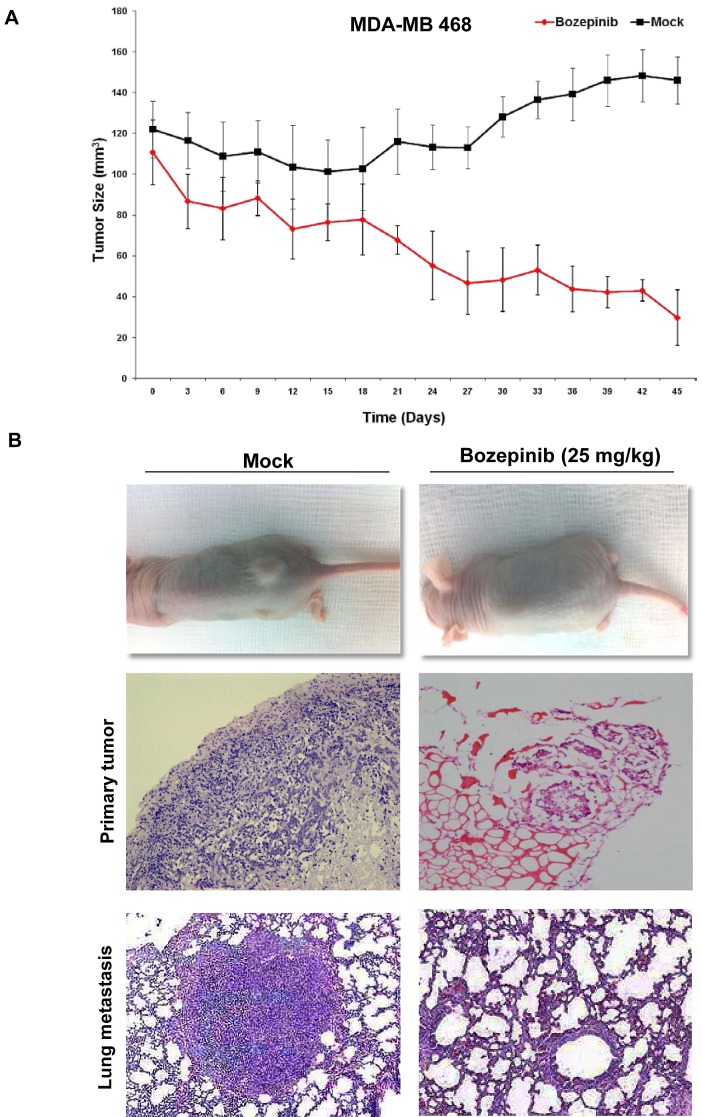
*In vivo* antitumor activity of Bozepinib. (A) *In vivo* determination of Bozepinib anti-tumor activity in MDA-MB 468 breast cancer cells. Mock-treated mice with vehicle alone (n= 8) and treated with Bozepinib (25mg/kg, n=8). (B) Representative hematoxylin and eosin (H&E)-stained sections of tumors in mice mock-treated (10×) and treated with Bozepinib (4×) and representative H&E-stained lung section from a metastasic nodule (10×) in mock-treated mice and Bozepinib-treated mice (20×).

To evaluate the effect of Bozepinib on tumour growth, heterotopic tumour xenografts were established using the MDA-MB 468 breast cancer cell line and the HT-29 colon cancer cell line. Nine days later, tumors reached a size superior to 100 mm^3^, and the mice received an i.p. injection of 25 mg/kg of Bozepinib or methylcellulose 1%, respectively. Bozepinib treatment significantly inhibited colon and breast cancer tumor growth when compared with control group (P < 0.01, n = 12). From day 3 for HT-29 cell line and day 18 for MDA-MB 468 after Bozepinib injection, the average of tumor volume was significantly lower in comparison with control group (Fig. 6A-B and Supplementary Figure S2B). Inmunohistochemistry analysis showed that primary breast cancer tumours had a different morphological appearance between mice mock-treated and treated with Bozepinib. Control group had a significant cell number indicative of a highly proliferative tissue; however, mice treated with Bozepinib displayed small size of tumors and with necrotic and non-viable tumor areas (Fig. 6B). The moderate ability of MDA-MB 468 cells to form metastasis in nude mice has been described [23]. We were able to detect lung metastasis in 5 of 6 (83.3%) mice in the control group (Fig. 6B) and, surprisingly, only 1 out of 6 mice (16.6%) treated with Bozepinib presented a lung metastasis. The antimigratory effect of Bozepinib together these *in vivo* results suggest the potential of Bozepinib also as an anti-metastatic compound.

## DISCUSSION

Identification and characterization of new pharmacological activities from novel drugs represent an effective way of accelerating the translation of discoveries from bench to clinical applications. Previously, we have identified Bozepinib as a potent anti-tumor compound against breast and colon cancer cells [17, 18, 24]. Although we described the role of protein kinase PKR as a target of Bozepinib involved in the apoptosis of breast and colon cancer cells [18], here we have made a profound study of the mechanism of action of this drug. We demonstrated the selective effect of Bozepinib that exhibited high TI in both breast and colon cancer cells, and we identified interesting signaling pathways involved in its efficacy.

Bozepinib was a selective inhibitor of HER2 positive breast cancer cells as we demonstrated by the kinase assay, also verified with immunoblot analysis in the HER2 positive SKBR-3 breast cancer cell line, and in the xenotrasplant of HER 2 positive HT-29 colon cancer cells [25] in nude mice. Approximately, 25-30% of all primary breast tumors over-express the HER2 receptor, which is associated with a poor prognosis and an overall survival decrease in patients [5]. Trastuzumab, is actually used in first line clinical treatment for metastatic HER2 positive breast tumors, showing an overall survival improvement in patients. However, a considerable percentage of patients with metastatic disease ultimately develop resistance to trastuzumab [26], which increases the need of developing novel targeted therapies that overcome treatment resistance. In this way it is important to target not only the proliferating related receptors but also downstream signaling proteins involved in this process. In fact, current therapies are based on the combinations of different drugs with the aim to target both HER2 receptor and downstream signaling pathways such as tyrosine kinase inhibitors and mTOR inhibitors among others [27]. Bozepinib also presented an inhibitory effect over AKT, JNK, ERKs and VEGF signaling pathways. AKT is an important part of PI3K signaling and its activation is involved in tumor progression through increased cell proliferation, invasion and angiogenesis [28]. More investigation is necessary in order to determine if the inhibition of AKT phosphorylation induced by Bozepinib would be involved in the down-regulation of constitutively active PI3K/Akt/mTOR. Whereas an inhibition on EGFR activity was detected by the kinome assay, we were not able to detect changes in the phosphorylation of the receptor in EGFR positive MDA-MB 468 cells treated with EGF and Bozepinib (data not shown). However, we detected inhibition in the JNK and ERKs phosphorylation in both MCF-7 cells described as negative for EGFR expression and in MDA-MB 468 cells expressing high EGFR levels [29]. ERK inhibitors are currently considered as a therapeutic option for the treatment of patients who relapse on BRAF or MEK inhibitor therapy [30]. The selective effect of Bozepinib over tumors expressing these mutations opens a new via to be investigated with this novel drug. The therapeutic effect of JNK inhibitors has been described as proliferation suppressors with anti-tumor consequences and, in fact, several JNK inhibitors are currently in preclinical stages [31, 32]. Our data suggest that Bozepinib is a selective inhibitor of HER2 signaling, but its anti-tumor activity on breast and colon cancer cells lines expressing different receptors patterns suggests several mechanisms of action for this compound.

The inhibition of proliferative signaling usually converges in the inhibition of angiogenesis and cell migration [31]. In fact, Bozepinib was able to reduce the VEGF basal level detected in the SKBR-3 cancer cells and showed inhibitory effects against VEGFR-1, VEGFR-2 and VEGFR-3 in the kinome assay. In accordance with the anti-angiogenic properties and the inhibition of migration presented by Bozepinib in cancer cell lines, the metastatic capacity of MDA-MB 468 cells xenotransplanted in nude mice was also inhibited after i.p. treatment, suggesting the clinical potential of this promising anti-tumor drug.

The modulation of pathways involved in tumorigenesis by Bozepinib was also evident in the gene-profiling assays carried out by microarrays analysis in breast cancer-treated cells at different times. The data obtained suggest numerous mechanisms that could be involved in the anti-tumor activity of this compound. Bozepinib treatment up-regulated several genes involved in metabolic routes, such as cytochrome p450 family genes and glutathione metabolism related genes. Both routes are not only involved in the metabolism of drugs but also in anti-tumor processes such as detoxification, stress response, apoptosis and proliferation [33, 34]. Moreover, the gene expression of Cysthiathionine-Lyase CSE, an enzyme with anti-proliferative properties that engages ERKs and JNK signaling [35], was up-regulated after Bozepinib treatment. In addition, we found the up-regulation of genes as the chemokine *CXCL10*, involved in immune and inflammatory processes and that posses anti-angiogenic activity, which are associated with increased immune infiltration and improved survival in patients with solid malignancies including breast cancer [36, 37]. On the other hand, genes involved in breast cancer tumorigenesis such as *CLAUDIN1* and *E2F8* among others, were down-regulated after Bozepinib-treatment. It has been previously suggested that *CLAUDIN 1* acts as a tumor suppressor. However, recent studies have shown that this protein participates directly in promoting breast cancer progression, possibly through the alteration of the expression of epithelial-mesenchymal transition genes, and actually its down-regulation has been described as a contribution to the inhibition of breast cancer migration [38]. The deregulation of the E2F family member of transcription factors including the E2F8 atypical member, contributes to oncogenesis and progression. Ectopic over-expression of E2F8 promoted cell proliferation, colony formation and tumorigenicity, whereas the E2F8 knockdown inhibited these phenotypes [39] suggesting that the down-regulation of E2F8 induced by Bozepinib could contribute to its anti-tumor effect. Moreover, it has recently been shown that HER2 tumorigenesis and metastasis are regulated by E2F activator transcription factors [40].

Surprisingly, Bozepinib induced the down-regulation of important genes involved in Notch and Wnt signaling. These genes contribute to the CSC phenotype when they are de-regulated [41]. We detected a down-regulation of the co-activator Mastermind-like MAML-2 protein, which decreases Notch signaling reducing the primary tumor sphere formation and side population in MCF-7 cell line, contributing to the decrease in the number of CSC subpopulations [42]. We also found an evident down-regulation of the *NOTCH 3* gene, which has recently been involved in the proliferation of both HER2 positive and negative breast cancer cells, suggesting that targeted suppression of this signaling pathway may be a promising strategy for the treatment of determined HER2-related breast cancers [43, 44]. According to the proteomic and genomic results obtained after Bozepinib treatment and considering the recent works relating HER2 positivity with CSC phenotype in breast cancer cells with high ALDH activity [45, 46], we decided to analyze the efficacy of Bozepinib over CSCs enriched subpopulations. Since there is controversy about the efficacy of sphere-forming cultures to select CSCs [47, 48] we used ALDH activity to enrich cell cultures with clonal ability and stem-like properties. Moreover, previous to the treatment with Bozepinib the *in vivo* tumorigenic potential of ALDH+ and the reestablishment of tumor heterogeneity was demonstrated (data not shown). Our results showed that Bozepinib inhibited both mamo- and colono-spheres formation in subpopulations isolated by ALDH activity. High ALDH activity is associated with metastasis, resistance to chemotherapies and poor prognosis in human cancers, and it has been identified as one of the most specific markers of human CSCs [49]. Moreover, Bozepinib was able to induce apoptosis in the resistant ALDH + subpopulations from both SKBR-3 and HCT-116 cells, although in less degree that the more sensible ALDH– subpopulations. CSCs are apoptosis resistant in order to secure the production of progeny [50] and an apoptosis program is a prerequisite for any tumor-initiating cell and may support the survival of CSCs in response to chemo- or radio-therapy. Therefore, targeted therapies reactivating death program and inducing apoptosis in CSCs may synergize with established therapies and increase efficacy in the clinic. In addition, Bozepinib displayed IC_50_ values in a similar range to Salinomycin, which has been described as a selective and potent drug against CSCs with limited use in human due to considerable toxicity [21]. Interestingly, we detected a significant reduction in the level of c-MYC and β-CATENIN in both breast and colon ALDH+ and ALDH- cells after Bozepinib treatment. Although most of these proteins are expressed in normal and differentiated tumor cells, it has been described that the expression level of determined genes involved in stem-like properties determines the CSCs process [51]. In accordance with the high level of stem signaling proteins described for CSCs, we detected higher levels of these proteins in the ALDH+ subpopulations in comparison with ALDH- cells. Recently, it has been reported the cooperation between c-MYC and HER2 and between c-MYC and β-CATENIN to drive mammary stem cell amplification and tumorigenesis [10, 12]. In fact, the down-regulation of c-MYC triggers the inhibition of cancer cell proliferation, invasion and migration [52]. Wnt/β-catenin signaling plays a critical role in CSCs, being β-CATENIN a key mediator of Wnt signaling [53]. Moreover, whereas no detectable levels of SOX2 protein were present in breast cancer cells, we detected a considerable level of SOX2 in ALDH-isolated colon cancer cells that was significantly reduced after Bozepinib treatment. The transcription factor SOX2 is involved together with c-MYC, KLF4 and OCT3/4 in the induction and maintenance of pluripotent stem cells and has been also associated the expression of both SOX2 and β-CATENIN with metastases and poor prognosis in colon cancer [15]. Finally, we detected significant changes in GLI-3 expression in colon cancer cells, a described target gene transcription repressor of Hedgehog signaling pathway [22]. Whereas GLI-3 expression was detected in ALDH- HCT-116 isolated cells, this protein was not detected in the ALDH+ cells, suggesting that the Hedgehog signaling pathway is involved in the CSC phenotype as has been previously described [22]. The treatment with Bozepinib induced a strong expression of GLI-3 protein level in both ALDH+ and ALDH- colon cancer cells. In addition, according to reported data [54], in our study, the activation of GLI-3 protein corresponded with an inactivation of β-CATENIN expression in CSCs subpopulations after Bozepinib treatment. Since *GLI-3* over-expression reduced tumor cell proliferation and induced apoptosis in colon CSCs [55], the GLI-3 induction by Bozepinib could be one of the mechanisms by which this drug exerts its anti-tumor activity in colon CSCs that must be deeply explored.

Although we show promising data proving the efficacy of Bozepinib over CSCs, the mechanism by which Bozepinib inhibits the CSC growth requires further detailed investigation. The Bozepinib effect involves different pathways and kinases as we described in a previous work showing the role of the PKR pro-apoptotic kinase [18], and in the present study by the inhibition of proliferative signaling pathways. However, the determination of the impact of every kinase or every signaling pathway of the drug is not yet known and probably it is a pleiotropic effect that finally leads to its effectiveness. Moreover, the specific HER2, JNK and ERKs inhibition, the anti-angiogenic and anti-migration activity together with the *in vivo* anti-tumor and anti-metastatic effect and the non-systemic toxicity of Bozepinib, encourage further studies on the therapeutic potential of this novel synthetic compound in breast and colon cancer patients.

## MATERIALS AND METHODS

### Drugs and treatments

(*RS*)-2,6-Dichloro-9-[1-(*p*-nitrobenzenesulfonyl)-1,2,3,5-tetrahydro-4,1-benzoxazepin-3-yl]-9*H*-purine (Bozepinib) was synthesized as previously described [17], dissolved in DMSO and stored at −20 °C. For each experiment, the stock solutions were further diluted in medium to obtain the desired concentrations. The final solvent concentration in cell culture was ≤ 0.1% v/v of DMSO, a concentration without effect on cell replication. Parallel cultures of cells in medium with DMSO were used as controls.

### Cell lines

The four human breast cancer cell lines MCF-7, MDA-MB-468, MDA-MB 231, SKBR-3 and the five human colon cancer cell lines Caco-2, T-84, HT-29, SW-480, HCT-116 were obtained from American Type Culture Collection (ATCC) and maintained in Dulbecco's Modified Eagle Medium (DMEM; Sigma-Aldrich) supplemented with 10% FBS. Non-tumoral cell lines MCF-10A (breast) and CCD-18Co (colon) obtained from ATCC were cultured in DMEM/F12 medium supplemented with 5% horse serum (HS), 0.5 μg/ml hydrocortisone, 0.02 μg/ml epithelial growth factor (EGF), 0.01 μg/ml insulin and 100ng/ml cholera toxin. HUVEC were cultured in endothelial growth medium (EGM-2; Lonza). All cell lines listed above were tested for authentication using the short-tandem repeat profiling and were passaged for less than 6 months, and routinely assayed for mycoplasma contamination.

### *In vitro* cytotoxicity assays

The effect of anticancer drugs on cell viability was assessed using the sulforhodamine-B colorimetric assay. Briefly, cells (1×10^3^ cells/well) were seeded onto 24-well plates and incubated for 24 h and then treated with different Bozepinib concentrations. Three days later, wells were aspirated, fresh medium and drug were added, and cells were maintained for 3 additional days. Cells were maintained with the drug for six days. Thereafter, cells were processed as previously described [56], using a Titertek Multiscan apparatus (Flow, Irvine, California) at 492 nm. We evaluated the linearity of the SRB assay with a cell number for each cell line before every cell growth experiment. The IC_50_ values were calculated from semi-logarithmic dose-response curves by linear interpolation. All of the experiments were plated in triplicate wells and were carried out at least twice.

### Kinase inhibition assays

The kinase inhibition profile of Bozepinib was determined using a panel of 36 protein kinases (Supplementary Table S1). Residual activity values were measured by testing each compound at two concentrations (5×10^−5^ M and 5×10^−6^ M) in singlicate for each kinase assay. Detailed methods for kinome analyses are provided in Supplementary Materials and Methods.

### MTS cell viability assay

Enriched subpopulations of CSCs were seeded in a concentration of 3000 cells/well in 96-well plates in sphere forming medium and treated with different Bozepinib concentrations. After 72 h, 10 μl of CellTiter 96® AQueous One Solution Cell Proliferation Assay, MTS (10 mg/ml) (Promega Corporation, Madison, USA) was added to each well and incubated at 37°C for 2-4 h. Plates were read at 570 nm on a Bio-Rad plate reader.

### Apoptosis analysis

Cells were plated in six-well plates and after treatment were trypsinized and analyzed using an Annexin V-fluorescein isothiocyanate detection kit (eBioscience Inc., San Diego, CA, USA). The samples were immediately processed using a FACSAria III flow cytometer (Becton Dickinson, BD Biosciences, Franklin Lakes, NJ, USA) from the Scientific Instrumental Center (University of Granada).

### Western blotting

For attached cell lines, cells were seeded on 6-well plates in their respective medium. After treatment, medium was removed from attached cells and floating CSCs spheres were centrifugated at 1500 rpm, washed twice with PBS and then lysed in Laemmli buffer. Immunoblotting on whole cell lysates was performed following routine protocols [18]. All antibodies used for western blot analysis are listed in the Supplementary Material and Methods.

### Functional capillary formation assays

The ability of Bozepinib to inhibit capillaries in semisolid medium was tested in HUVEC using a Matrigel assay (see Supplementary Material and Methods).

### Wound-healing assay

Breast and colon cancer cells were seeded in 6-well plates and grown to 80% confluence. Wounds were created by scraping monolayer cells with a 200 μl (for HCT-116) and 1000 μl (for SKBR-3) pipette tip, and non-adherent cells were washed off with medium. At 0, 24 and 48 h after the creation of wounds, Bozepinib-treated and control non-treated cells were observed and photographed with a 10× objective in a phase-contrast microscopy. Wound distances were measured at each time point and expressed as the average percent of wound closure by comparing the zero time. Image-Pro Plus 6.0 software was used to quantify the wound area. All experiments were plated in triplicate wells and were carried out at least three times.

### Microarray analysis

MDA-MB 468 cells were treated with 5 μM Bozepinib for 4 h and 16 h. No treated cells were used as control. Total RNAs were extracted using Quiagen extraction Kit, according to the manufacturer's protocol. RNA microarray analyses were carried out with the Affymetrix Human Gene 1.0 ST arrays according to the Affymetrix standard protocol. The gene list with x-fold cut-off was then subdivided into functional categories with the bioinformatic analysis resource DAVID (NCI, Frederick, http://david.abcc.ncifcrf.gov). Gene pathway analysis was also conducted by DAVID and Ingenuity Pathways Analysis (IPA, https://www.ingenuity.com/).

### Quantitative real time RT-PCR

Total RNA from the different cell lines was extracted from duplicate 80% confluent cultures using the TRIZOL reagent following the instructions of the manufacturer (Life Technologies). cDNA was synthesized by reverse transcrition of total RNA using the Reverse Transcription System (Promega) and qRT-PCR assay was done using SYBR Green PCR Master Mix (Promega) and random primers. Each reaction was performed in triplicate from two cDNA dilutions. The comparative threshold cycle (Ct) method was used to calculate the amplification factor as specified by the manufacturer. Human GADPH was used as an internal standard to normalize variations in RNA quality in the quantities of input cDNA. The amount of target and endogenous reference was determined from a standard curve for each experimental sample. The standard curve was constructed by 5-fold serial dilutions of cDNA from 1 μg. Primer sequences are listed in Supplementary Table S2.

### Isolation of CSCs

Cancer stem-like cells from SKBR-3 breast and HCT-116 colon cancer cells lines were isolated using the ALDEFLUOR assay (StemCell Technologies) by fluorescence-activated cell sorting (FACS) (see Supplementary Material and Methods) and enriched subpopulation of CSCs were grown with a specific sphere-forming medium in ultralow attachment plates (Corning) as previously described [49].

### *In vivo* sub-acute toxicity and anti-tumor xenograft studies

Sub-acute toxicity was determined in six weeks old BALBC/c female (n=20) during 29 days. Bozepinib dissolved in methylcellulose 1% was administered in a single i.p. bolus injection at 100 mg/kg twice a week. Control mice were (n=10) inoculated with the same volume of methylcellulose. Mice were maintained under standard conditions and for each treatment schedule, were weighed and assessed twice weekly for systemic toxicity (listlessness, weight loss) and local toxicity (alopecia, skin reaction, and leg motility).

To establish xenograft tumors six- to eight-week old female BALB/c nude mice (Charles Rives Laboratories) were used. All procedures were approved by the Institutional Animal Care and Use Committee at the University of Granada. They were housed and maintained at 20°C to 24°C, 50% RH, a 14 to 10 h light-dark cycle with food and water ad libitum. The HT-29 and MDA-MB 468 tumors were generated by subcutaneous injections of 2×10^6^ cells/mouse using 26-gauge needles. Tumors were allowed to grow to an average volume of 100 mm^3^. Animals (n= 8 per group) were then randomly assigned as control and treatment groups and treated with methylcellulose 1% (control vehicle) or Bozepinib (25 mg/kg, dissolved in methylcellulose 1%) injected i.p. three times a week for 45 days for MDA-MB 468 and two weeks for HT-29. Tumor weight was calculated according to the formula: TW (mg) = tumor volume (mm^3^) = d^2^ × D/2, where d and D are the shortest and longest diameters, respectively. Paraffin-embedded blocks of all tumors were sectioned at 5 μm. Each sample was stained with hematoxylin and eosin (H&E) for histopathologic analysis.

### Statistical analysis

Data are presented as mean ± SD. Differences between groups for each sample were tested by one-way ANOVA. Assumptions of analysis of variance (homocedesticity and normality) were tested and assured by using transformed data sets [log (dependent variable value 1] when necessary. Significance was accepted at P < 0.05 in all cases.

## SUPPLEMENTARY MATERIALS AND METHODS FIGURES AND TABLES



## References

[1] Jemal A, Bray F, Center MM, Ferlay J, Ward E, Forman D (2011). Global cancer statistics. CA Cancer J Clin.

[2] Hendriks BS, Opresko LK, Wiley HS, Lauffenburger D (2003). Coregulation of epidermal growth factor receptor/human epidermal growth factor receptor 2 (HER2) levels and locations: quantitative analysis of HER2 overexpression effects. Cancer Res.

[3] Yarden Y (2001). The EGFR family and its ligands in human cancer. signalling mechanisms and therapeutic opportunities. Eur J Cancer.

[4] Duffy MJ, Lamerz R, Haglund C, Nicolini A, Kalousova M, Holubec L, Sturgeon C (2013). Tumor markers in colorectal cancer, gastric cancer and gastrointestinal stromal cancers: European group on tumor markers 2014 guidelines update. Int J Cancer.

[5] Puhalla S, Brufsky A (2013). Treatment of HER2-positive breast cancer: looking backwards briefly. Lancet Oncol.

[6] O'Brien Z, Fallah Moghaddam M (2013). Small molecule kinase inhibitors approved by the FDA from 2000 to 2011: a systematic review of preclinical ADME data. Expert Opin Drug Metab Toxicol.

[7] Knight ZA, Lin H, Shokat KM (2010). Targeting the cancer kinome through polypharmacology. Nat Rev Cancer.

[8] Bonnet D, Dick JE (1997). Human acute myeloid leukemia is organized as a hierarchy that originates from a primitive hematopoietic cell. Nat Med.

[9] Visvader JE, Lindeman GJ (2008). Cancer stem cells in solid tumours: accumulating evidence and unresolved questions. Nat Rev Cancer.

[10] Nair R, Roden DL, Teo WS, McFarland A, Junankar S, Ye S, Nguyen A, Yang J, Nikolic I, Hui M, Morey A, Shah J, Pfefferle AD, Usary J, Selinger C, Baker LA (2013). c-Myc and Her2 cooperate to drive a stem-like phenotype with poor prognosis in breast cancer. Oncogene.

[11] Sussman RT, Ricci MS, Hart LS, Sun SY, El-Deiry WS (2007). Chemotherapy-resistant side-population of colon cancer cells has a higher sensitivity to TRAIL than the non-SP, a higher expression of c-Myc and TRAIL-receptor DR4. Cancer Biol Ther.

[12] Moumen M, Chiche A, Decraene C, Petit V, Gandarillas A, Deugnier MA, Glukhova MA, Faraldo MM (2013). Myc is required for beta-catenin-mediated mammary stem cell amplification and tumorigenesis. Mol Cancer.

[13] Tenbaum SP, Ordonez-Moran P, Puig I, Chicote I, Arques O, Landolfi S, Fernandez Y, Herance JR, Gispert JD, Mendizabal L, Aguilar S, Ramon y Cajal S, Schwartz S, Vivancos A, Espin E, Rojas S (2012). beta-catenin confers resistance to PI3K and AKT inhibitors and subverts FOXO3a to promote metastasis in colon cancer. Nat Med.

[14] Leis O, Eguiara A, Lopez-Arribillaga E, Alberdi MJ, Hernandez-Garcia S, Elorriaga K, Pandiella A, Rezola R, Martin AG (2012). Sox2 expression in breast tumours and activation in breast cancer stem cells. Oncogene.

[15] Neumann J, Bahr F, Horst D, Kriegl L, Engel J, Luque RM, Gerhard M, Kirchner T, Jung A (2011). SOX2 expression correlates with lymph-node metastases and distant spread in right-sided colon cancer. BMC Cancer.

[16] Deng S, Yang X, Lassus H, Liang S, Kaur S, Ye Q, Li C, Wang LP, Roby KF, Orsulic S, Connolly DC, Zhang Y, Montone K, Butzow R, Coukos G, Zhang L (2010). Distinct expression levels and patterns of stem cell marker, aldehyde dehydrogenase isoform 1 (ALDH1), in human epithelial cancers. PLoS One.

[17] Lopez-Cara LC, Conejo-Garcia A, Marchal JA, Macchione G, Cruz-Lopez O, Boulaiz H, Garcia MA, Rodriguez-Serrano F, Ramirez A, Cativiela C, Jimenez AI, Garcia-Ruiz JM, Choquesillo-Lazarte D, Aranega A, Campos JM (2011). New (RS)-benzoxazepin-purines with antitumour activity: The chiral switch from (RS)-2,6-dichloro-9-[1-(p-nitrobenzenesulfonyl)-1,2,3,5-tetrahydro-4,1-benzoxazep in-3-yl]-9H-purine. Eur J Med Chem.

[18] Marchal JA, Carrasco E, Ramirez A, Jimenez G, Olmedo C, Peran M, Agil A, Conejo-Garcia A, Cruz-Lopez O, Campos JM, Garcia MA (2013). Bozepinib, a novel small antitumor agent, induces PKR-mediated apoptosis and synergizes with IFNalpha triggering apoptosis, autophagy and senescence. Drug Des Devel Ther.

[19] Wang SE, Yu Y, Criswell TL, Debusk LM, Lin PC, Zent R, Johnson DH, Ren X, Arteaga CL (2010). Oncogenic mutations regulate tumor microenvironment through induction of growth factors and angiogenic mediators. Oncogene.

[20] von Marschall Z, Scholz A, Cramer T, Schafer G, Schirner M, Oberg K, Wiedenmann B, Hocker M, Rosewicz S (2003). Effects of interferon alpha on vascular endothelial growth factor gene transcription and tumor angiogenesis. J Natl Cancer Inst.

[21] Naujokat C, Steinhart R (2012). Salinomycin as a drug for targeting human cancer stem cells. J Biomed Biotechnol.

[22] Takebe N, Harris PJ, Warren RQ, Ivy SP (2011). Targeting cancer stem cells by inhibiting Wnt, Notch, and Hedgehog pathways. Nat Rev Clin Oncol.

[23] Gorges TM, Schiller J, Schmitz A, Schuetzmann D, Schatz C, Zollner TM, Krahn T, von Ahsen O (2012). Cancer therapy monitoring in xenografts by quantitative analysis of circulating tumor DNA. Biomarkers.

[24] Nunez MC, Diaz-Gavilan M, Conejo-Garcia A, Cruz-Lopez O, Gallo MA, Espinosa A, Campos JM (2008). Design, synthesis and anticancer activity against the MCF-7 cell line of benzo-fused 1,4-dihetero seven- and six-membered tethered pyrimidines and purines. Curr Med Chem.

[25] Pohl M, Stricker I, Schoeneck A, Schulmann K, Klein-Scory S, Schwarte-Waldhoff I, Hasmann M, Tannapfel A, Schmiegel W, Reinacher-Schick A (2009). Antitumor activity of the HER2 dimerization inhibitor pertuzumab on human colon cancer cells in vitro and in vivo. J Cancer Res Clin Oncol.

[26] Nahta R, Esteva FJ (2006). HER2 therapy: molecular mechanisms of trastuzumab resistance. Breast Cancer Res.

[27] Nielsen DL, Kumler I, Palshof JA, Andersson M (2013). Efficacy of HER2-targeted therapy in metastatic breast cancer. Monoclonal antibodies and tyrosine kinase inhibitors. Breast.

[28] Kumar A, Rajendran V, Sethumadhavan R, Purohit R (2013). AKT kinase pathway: a leading target in cancer research. ScientificWorldJournal.

[29] Kao J, Salari K, Bocanegra M, Choi YL, Girard L, Gandhi J, Kwei KA, Hernandez-Boussard T, Wang P, Gazdar AF, Minna JD, Pollack JR (2009). Molecular profiling of breast cancer cell lines defines relevant tumor models and provides a resource for cancer gene discovery. PLoS One.

[30] Morris EJ, Jha S, Restaino CR, Dayananth P, Zhu H, Cooper A, Carr D, Deng Y, Jin W, Black S, Long B, Liu J, Dinunzio E, Windsor W, Zhang R, Zhao S (2013). Discovery of a novel ERK inhibitor with activity in models of acquired resistance to BRAF and MEK inhibitors. Cancer Discov.

[31] Ennis BW, Fultz KE, Smith KA, Westwick JK, Zhu D, Boluro-Ajayi M, Bilter GK, Stein B (2005). Inhibition of tumor growth, angiogenesis, and tumor cell proliferation by a small molecule inhibitor of c-Jun N-terminal kinase. J Pharmacol Exp Ther.

[32] Takahashi R, Hirata Y, Sakitani K, Nakata W, Kinoshita H, Hayakawa Y, Nakagawa H, Sakamoto K, Hikiba Y, Ijichi H, Moses HL, Maeda S, Koike K (2013). Therapeutic effect of c-Jun N-terminal kinase inhibition on pancreatic cancer. Cancer Sci.

[33] Bernhardt R (2006). Cytochromes P450 as versatile biocatalysts. J Biotechnol.

[34] Wu Y, Fan Y, Xue B, Luo L, Shen J, Zhang S, Jiang Y, Yin Z (2006). Human glutathione S-transferase P1-1 interacts with TRAF2 and regulates TRAF2-ASK1 signals. Oncogene.

[35] Yang G, Cao K, Wu L, Wang R (2004). Cystathionine gamma-lyase overexpression inhibits cell proliferation via a H2S-dependent modulation of ERK1/2 phosphorylation and p21Cip/WAK-1. J Biol Chem.

[36] Datta D, Flaxenburg JA, Laxmanan S, Geehan C, Grimm M, Waaga-Gasser AM, Briscoe DM, Pal S (2006). Ras-induced modulation of CXCL10 and its receptor splice variant CXCR3-B in MDA-MB-435 and MCF-7 cells: relevance for the development of human breast cancer. Cancer Res.

[37] Bronger H, Kraeft S, Schwarz-Boeger U, Cerny C, Stockel A, Avril S, Kiechle M, Schmitt M (2012). Modulation of CXCR3 ligand secretion by prostaglandin E2 and cyclooxygenase inhibitors in human breast cancer. Breast Cancer Res.

[38] Blanchard AA, Ma X, Dueck KJ, Penner C, Cooper SC, Mulhall D, Murphy LC, Leygue E, Myal Y (2013). Claudin 1 expression in basal-like breast cancer is related to patient age. BMC Cancer.

[39] Deng Q, Wang Q, Zong WY, Zheng DL, Wen YX, Wang KS, Teng XM, Zhang X, Huang J, Han ZG (2010). E2F8 contributes to human hepatocellular carcinoma via regulating cell proliferation. Cancer Res.

[40] Andrechek ER (2013). HER2/Neu tumorigenesis and metastasis is regulated by E2F activator transcription factors. Oncogene.

[41] Dontu G, Jackson KW, McNicholas E, Kawamura MJ, Abdallah WM, Wicha MS (2004). Role of Notch signaling in cell-fate determination of human mammary stem/progenitor cells. Breast Cancer Res.

[42] Wong NK, Fuller M, Sung S, Wong F, Karsan A (2012). Heterogeneity of breast cancer stem cells as evidenced with Notch-dependent and Notch-independent populations. Cancer Med.

[43] Pradeep CR, Kostler WJ, Lauriola M, Granit RZ, Zhang F, Jacob-Hirsch J, Rechavi G, Nair HB, Hennessy BT, Gonzalez-Angulo AM, Tekmal RR, Ben-Porath I, Mills GB, Domany E, Yarden Y (2012). Modeling ductal carcinoma in situ: a HER2-Notch3 collaboration enables luminal filling. Oncogene.

[44] Yamaguchi N, Oyama T, Ito E, Satoh H, Azuma S, Hayashi M, Shimizu K, Honma R, Yanagisawa Y, Nishikawa A, Kawamura M, Imai J, Ohwada S, Tatsuta K, Inoue J, Semba K (2008). NOTCH3 signaling pathway plays crucial roles in the proliferation of ErbB2-negative human breast cancer cells. Cancer Res.

[45] Korkaya H, Wicha MS (2013). HER2 and breast cancer stem cells: more than meets the eye. Cancer Res.

[46] Duru N, Fan M, Candas D, Menaa C, Liu HC, Nantajit D, Wen Y, Xiao K, Eldridge A, Chromy BA, Li S, Spitz DR, Lam KS, Wicha MS, Li JJ (2012). HER2-associated radioresistance of breast cancer stem cells isolated from HER2-negative breast cancer cells. Clin Cancer Res.

[47] Smart CE, Morrison BJ, Saunus JM, Vargas AC, Keith P, Reid L, Wockner L, Amiri MA, Sarkar D, Simpson PT, Clarke C, Schmidt CW, Reynolds BA, Lakhani SR, Lopez JA (2013). In vitro analysis of breast cancer cell line tumourspheres and primary human breast epithelia mammospheres demonstrates inter- and intrasphere heterogeneity. PLoS One.

[48] Ma L, Lai D, Liu T, Cheng W, Guo L (2010). Cancer stem-like cells can be isolated with drug selection in human ovarian cancer cell line SKOV3. Acta Biochim Biophys Sin (Shanghai).

[49] Charafe-Jauffret E, Ginestier C, Iovino F, Tarpin C, Diebel M, Esterni B, Houvenaeghel G, Extra JM, Bertucci F, Jacquemier J, Xerri L, Dontu G, Stassi G, Xiao Y, Barsky SH, Birnbaum D (2010). Aldehyde dehydrogenase 1-positive cancer stem cells mediate metastasis and poor clinical outcome in inflammatory breast cancer. Clin Cancer Res.

[50] Kruyt FA, Schuringa JJ (2010). Apoptosis and cancer stem cells: Implications for apoptosis targeted therapy. Biochem Pharmacol.

[51] Zardawi SJ, O'Toole SA, Sutherland RL, Musgrove EA (2009). Dysregulation of Hedgehog, Wnt and Notch signalling pathways in breast cancer. Histol Histopathol.

[52] Zhao Y, Jian W, Gao W, Zheng YX, Wang YK, Zhou ZQ, Zhang H, Wang CJ (2013). RNAi silencing of c-Myc inhibits cell migration, invasion, and proliferation in HepG2 human hepatocellular carcinoma cell line: c-Myc silencing in hepatocellular carcinoma cell. Cancer Cell Int.

[53] Reya T, Clevers H (2005). Wnt signalling in stem cells and cancer. Nature.

[54] Ulloa F, Itasaki N, Briscoe J (2007). Inhibitory Gli3 activity negatively regulates Wnt/beta-catenin signaling. Curr Biol.

[55] Merchant AA, Matsui W (2010). Targeting Hedgehog--a cancer stem cell pathway. Clin Cancer Res.

[56] Marchal JA, Boulaiz H, Suarez I, Saniger E, Campos J, Carrillo E, Prados J, Gallo MA, Espinosa A, Aranega A (2004). Growth inhibition, G(1)-arrest, and apoptosis in MCF-7 human breast cancer cells by novel highly lipophilic 5-fluorouracil derivatives. Invest New Drugs.

