# Supplementary material for: HER2-signaling pathway, JNK and ERKs kinases, and cancer stem-like cells are targets of Bozepinib
Source: Oncotarget. 2014 May 13;5(11):3590–606. doi: 10.18632/oncotarget.1962 (PMC4116505; doi:10.18632/oncotarget.1962)
Supplement: Supplementary file 1 [file oncotarget-05-3590-s001.pdf]

HER2-signaling pathway, JNK and ERKs kinases, and cancer stem-like cells are targets of Bozepinib small compound

MATERIAL AND METHODS

Aldefluor assay and separation of the ALDH1-positive cell population by FACS

The isolation of CSCs from SKBR-3, MDA-MB 468, and HCT-116 cell lines was carried out using the Aldefluor kit (Stem Cell Technologies), which detects the activity of aldehyde dehydrogenase (ALDH). The experiments were undertaken according to the manufacturer's instructions. Cells were suspended in ALDEFLUOR assay buffer containing ALDH substrate (BAAA, 1 µmol/l per 1×10^{6} cells) and incubated during 40 minutes at 37°C. As a negative control, for each sample of cells an aliquot was treated with 50 mmol/L of diethylaminobenzaldehyde (DEAB) (Sigma), a specific ALDH inhibitor. The brightly fluorescent ALDH1-expressing cells (ALDH1-positive cells) were detected in the green fluorescence channel (520-540 nm) of a FACScan Aria III. The sorting gates were established using as negative controls the cells stained with Propidium iodide (Sigma) only. Each experiment was repeated three times.

Kinase Inhibition Assays

Bozepinib was dissolved in volumes of 100% DMSO to reach stock concentrations of 5×10^{-3} M/100% DMSO and 5×10^{-4} M/100% DMSO, respectively. In the process, the 5×10^{-3} M/100% DMSO and 5×10^{-4} M/100% DMSO solutions were diluted with water to 5×10^{-4} M/10% DMSO and 5×10^{-5} M/10% DMSO in a 96 well microtiter plate directly before use. For every kinase, 5 µl from each of the 5×10^{-4} M/10% DMSO and 5×10^{-5} M/10% DMSO compound solutions were transferred into the assay plates. The

final assay concentration of the compounds was 5×10^{-5} M and 5×10^{-6} M in 1 % DMSO in a 50 µl reaction volume. Residual activity values were measured by testing each compound at two concentrations (5×10^{-5} M and 5×10^{-6} M) in singlicate in every kinase assay. All biochemical protein kinase activity assays were performed in 96-well FlashPlatesTM (Perkin Elmer, Boston, USA) in a 50 µl reaction volume. The reaction cocktail contained 25 µl of assay buffer /[γ-^{33}P]-ATP mixture, 5 µl of test compound (in 10% DMSO), 10 µl of substrate and 10 µl of purified recombinant protein kinase. Final concentration of ATP was 1 µM. The assay for all enzymes contained 60 mM HEPES-NaOH, pH 7.5, 3 mM MgCl2, 3 mM MnCl2, 3 µM Na-orthovanadate, 1.2 mM DTT, 50 µg/ml PEG20000, 1 µM ATP/[γ-^{33}P]-ATP (approx. 7×10^{05} cpm per well), protein kinase and substrate (variable amounts; see Table 1). The PKC-alpha assay additionally contained 1 mM $CaCl_2$, 4 mM EDTA, 5 µg/ml Phosphatidylserine and 1 µg/ml 1.2-Dioleyl-glycerol. The reaction cocktails were incubated at 30 °C for 1 h. The reaction was halted with 50 µl of 2% (v/v) H_3PO_4; plates were aspirated and washed twice with 200 µl 0.9% (w/v) NaCl. The incorporation of ^{33}Pi was determined with a microplate scintillation counter (Microbeta, Wallac The residual activity (in %) for each compound well was calculated by using the following formula:

Res. Activity (%) = 100 X [(cpm of compound – low control) / (high control – low control)]

Western blotting

The protein sample was subjected to electrophoresis, transferred onto nitrocellulose membranes (Bio-rad,162- 0115), and blocked in PBS containing 5% non-fat dry milk for 1 h at room temperature. Primary antibodies used included p-Her2 (Tyr1221/1222) (#2243), p-SAPK/JNK (Thr183/Tyr185) (#9255), p-ERK1/2 (Thr202/Tyr204) (#4377), p-AKT (Ser 473) (#9271) from Cell Signaling; HER-2 (sc-7301), AKT (sc-8312),

VEGF (sc-152), ERK1/2 (sc-292838), JNK (sc-571), GLI-3 (sc-74478), SOX-2 (sc-20088), c-MYC (sc-40), and β-CATENIN (sc-7963) from Santa Cruz and β-ACTIN (A2228) from Sigma-Aldrich. Secondary antibodies used included anti-rabbit IgG peroxidase conjugate (Sigma, A0545) and anti-mouse IgG peroxidase conjugate (Sigma, A9044). Protein–antibody complexes were visualized by enhanced chemiluminescence (ECL, Bonus, Amersham, Little Chalfont, UK) with the program IMAGE READER LAS-4000 in a LAS-4000 imaging system.

Functional capillary formation assays

The vessel-forming inhibition ability of Bozepinib was characterized by culturing HUVEC trypsinized cells on Matrigel™-coated 96-well plates (BD Biosciences) in EGM-2 medium. Matrigel™ was thawed, used to cover the culture plastic (50 µl per well of a 96-well plate) and allowed to solidify for 1 h at 37º C. Cells from stage I to stage IV cultures were independently seeded. Outgrowths obtained from cultures at different stages of the endothelial isolation process were seeded on Matrigel™-containing plates at 5 to 20×10^3 cells per well and cultured in EGM-2 medium for 7 days. Four hours, 24 h and 7 days after the initial plating photographs were taken using a Leica DM 5500B (Leica, Solms, Germany) microscope equipped with the Meta Systems software. Figures were processed with Adobe Photoshop 7.0. Cells were counted for the formation of capillary structures. The number of cord-like structures was measured after 24 h and each cord portion between the ramifications was considered one cord unit. Mean ± SE values were obtained by evaluating the whole cultures of each well under the same conditions from three independent experiments performed in duplicate. A semi-quantitative measurement of cord formation on Matrigel™ was developed (tube formation index) using HUVEC like control, as previously described [1, 2].

REFERENCES

1. Soares R, Balogh G, Guo S, Gartner F, Russo J and Schmitt F. Evidence for the notch signaling pathway on the role of estrogen in angiogenesis. Mol Endocrinol. 2004; 18(9):2333-2343.

2. Marchal JA, Picon M, Peran M, Bueno C, Jimenez-Navarro M, Carrillo E, Boulaiz H, Rodriguez N, Alvarez P, Menendez P, de Teresa E and Aranega A. Purification and long-term expansion of multipotent endothelial-like cells with potential cardiovascular regeneration. Stem Cells Dev. 2012; 21(4):562-574.

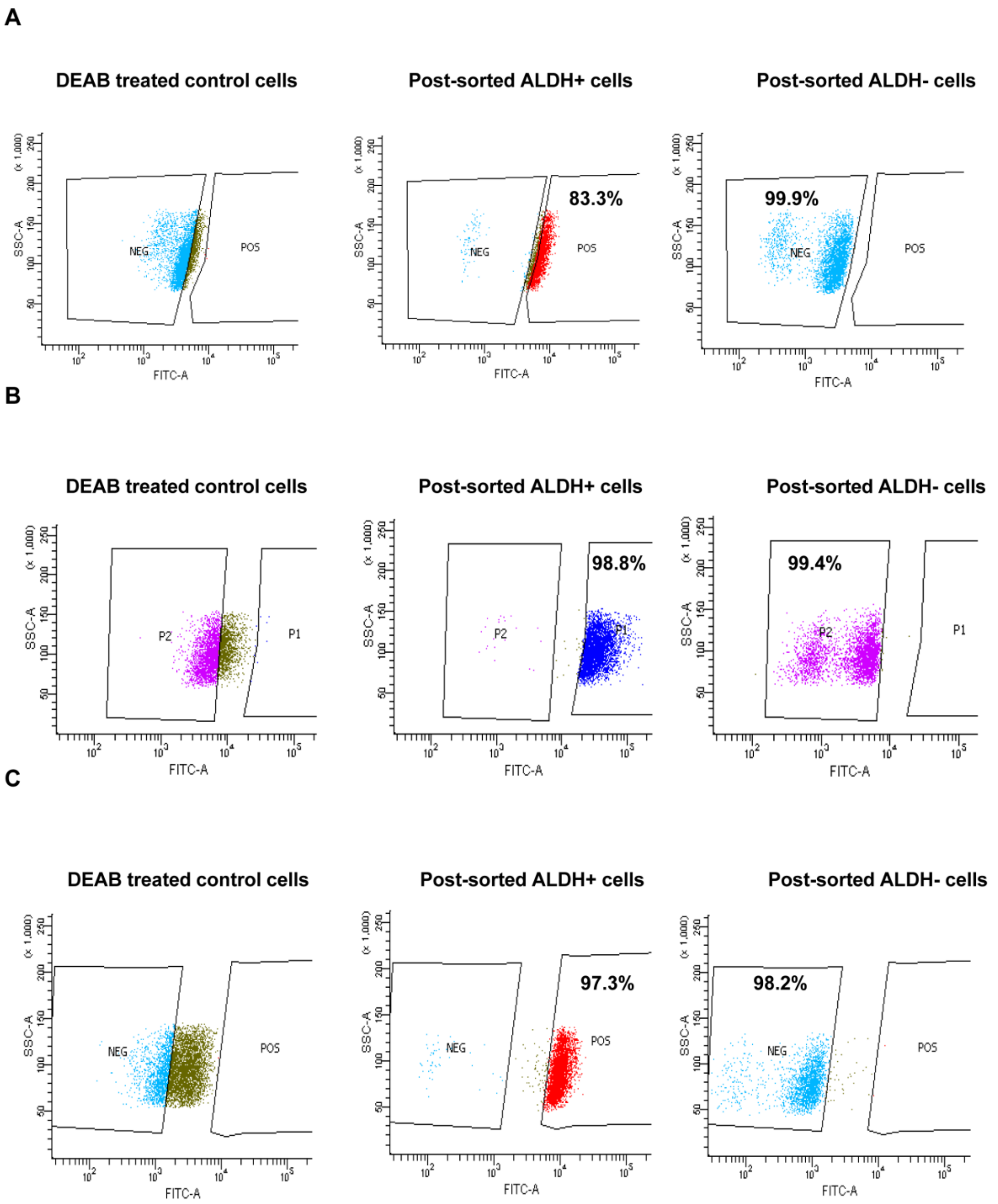

Figure S1: CSCs isolation using ALDH activity. Representative FACS analysis in SKBR-3 (**A**) and MDA-MB 468 (**B**) human breast and HCT-116 (**C**) colon cancer cell lines. Left panels represent DEAB treated control cells used to select ALDH+ and ALDH- populations to be sorted. Middle and right panels show the purity of ALDH+ and ALDH- populations, respectively, obtained after sorting.

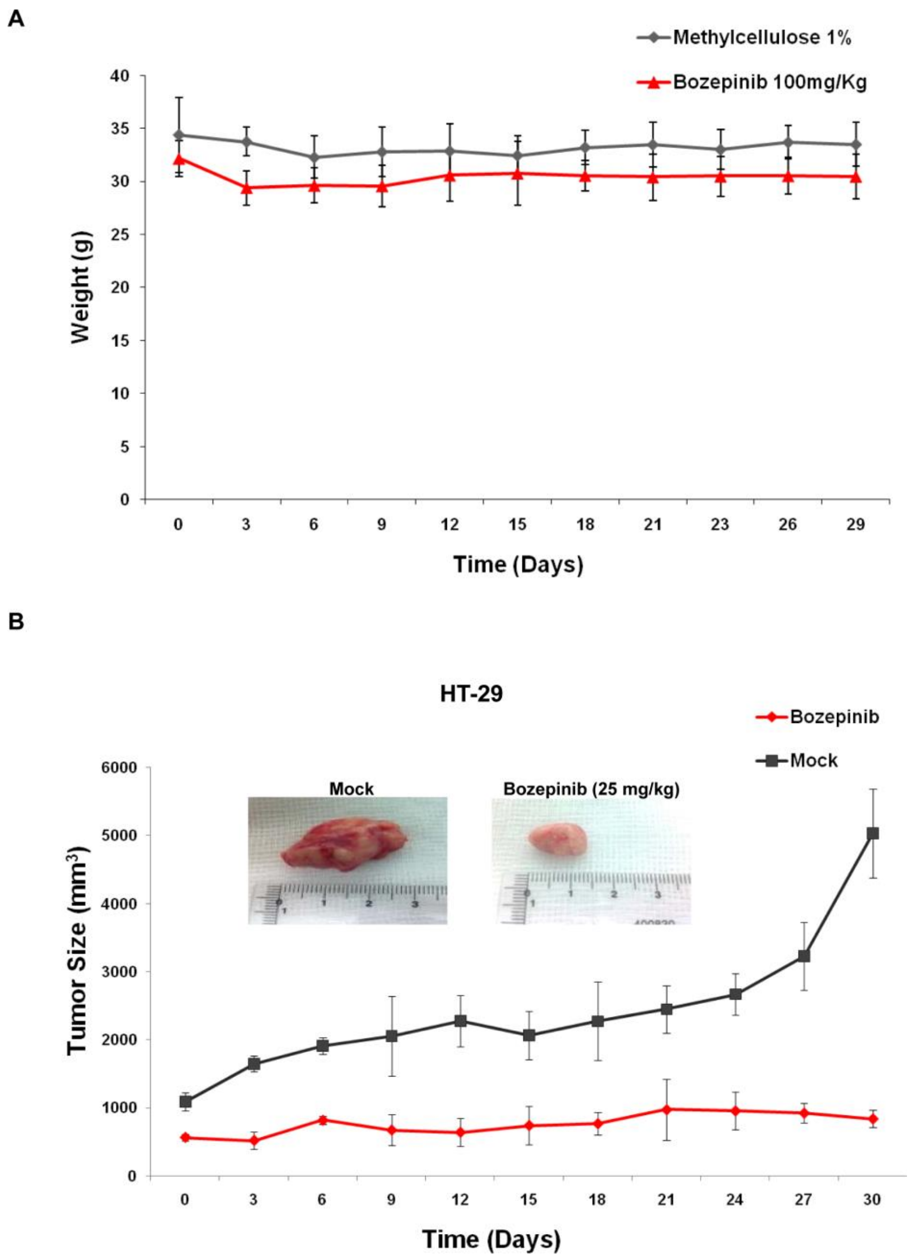

Figure S2: Bozepinib has *in vivo* antitumor activity without subacute toxicity. (A) Determination of subacute toxicity in BALB/c mice. Control mice (n=10) were treated with a solution of 1% metylcellulose. Bozepinib treated mice (n=10) were injected with a 100mg/kg dose. **(B)** Determination of Bozepinib antitumor activity in HT-29 colon cancer cells. Inserts represent tumor size of mock and Bozepinib treated mice.

**Table S1.** *Ex vivo* **kinome assay components. 36 kinases and the corresponding substrates are specified with the concentration assayed.**						
**No.**	**Kinase**	**Lot**	**Conc.(ng/50μl)**	**Substrate**	**Lot**	**Conc.(ng/50μl)**
**1**	AKT1	007	50	GSK3(14-27)	006	1.0
**2**	AKT2	003	200	GSK3(14-27)	006	1.0
**3**	AKT3	004	50	GSK3(14-27)	006	1.0
**4**	CDK/CycA	005	10	RBER-CHKtide	022	2.0
**5**	CDK/CycB1	024	25	MEK1-KM (Kinase-dead)	023	2.0
**6**	CDK/CycE	001	50	RBER-CHKtide	022	2.0
**7**	CDK/CycA	005	50	RBER-CHKtide	022.1	1.0
**8**	CDK/CycE	009	10	RBER-CHKtide	022.1	1.0
**9**	CDK/CycE	001	10	RBER-CHKtide	017	1.0
**10**	CDK/CycD1	007	25	RBER-CHKtide	022	2.0
**11**	CDK/CycD3	001	10	RBER-CHKtide	017	1.0
**12**	CDK/p25NCK	001	15	RBER-CHKtide	022	1.0
**13**	CDK/p35NCK	001	20	RBER-CHKtide	022.1	2.0
**14**	CDK6/CycD1	004	200	RBER-CHKtide	017	2.0
**15**	CDK7/CycH/MAT1	001	25	RBER-CHKtide	017	2.0
**16**	CDK8/CycC	002	50	RBER-IRStide	001	1.0
**17**	CDK9/CycT	004	15	RBER-CHKtide	022	1.0
**18**	EGF-R wt	012	10	Poly(Glu,Tyr)4.1	SIG_20K5903	0.125
**19**	ERBB2	004	100	Poly(Glu,Tyr)4.1	SIG_20K5903	0.125
**20**	ERK2	003	10	RBER-CHKtide	017	2.0
**21**	IKK- alpha	005	50	RBER-CHKtide	023	2.0
**22**	IKK-beta	006	100	RBER-CHKtide	017	1.0
**23**	IKK-epsilon	006	20	GSK3(14-27)	006	1.0
**24**	JAK3	002	200	Poly(Ala,Glu,Lys,Tyr)6:2:5:1	SIG_53H5516	0.125
**25**	JNK1	005	5	ATF2	006	0.5
**26**	JNK2	003	5	ATF2	006	0.25

**27**	JNK3	004	5	ATF2	006	0.25
**28**	PDK1	002	20	Tetra(LRRWSLG)	004	0.5
**29**	PKC-alpha	005	2,5	PKC(19-31)	002	0.25
**30**	P38-alpha	005	20	ATF2	006	0.5
**31**	RET	001	20	Poly((Glu,Tyr)4:1	SIG_20K5903	0.125
**32**	STK23	001	25	RBER-CHKtide	017	4.0
**33**	STK33	001	50	RBER-CHKtide	017	2.0
**34**	VEGF-R1	009	50	Poly(Glu,Tyr)4.1	SIG_20K5903	0.125
**35**	VEGF-R2	015	50	Poly(Glu,Tyr)4.1	SIG_20K5903	0.125
**36**	VEGF-R3	011	100	Poly(Glu,Tyr)4.1	SIG_20K5903	0.125

Table S2. qRT-PCR primer sequences

**Gene**	**Primer Sequence**	
CLAUDIN1	Forward	5´ GCGCGATATTTCTTCTTGCAGG-3´
	Reverse	5´TTCGTACCTGGCATTGACTGG-3´
CSE	Forward	5´ AGCCTTCATAATAGACTTCG 3´
	Reverse	5´ CAGCCCAGGATAAATAAC
CXCL10	Forward	5′ CCAGAATCGAAGGCCATCAA 3′
	Reverse	5′ CATTTCCTTGCTAACTGCTTTCAG 3′
E2F8	Forward	5´ GTGGATTACCTGAGGCCAAA 3´
	Reverse	5´ CTTCGTCAAGGCAGATGTCA 3´
MAML2	Forward	5´ ACATTTGTCAAGGCCACCTC 3´
	Reverse	5´ GTTTGCCAAAGCCTGGTTAG 3´
NOTCH3	Forward	5´ TGACCGTACTGGCGAGACT 3´
	Reverse	5´ CCGCTTGGCTGCATCAG 3´

Table S3. ***Ex vivo*** **inhibitory effect of Bozepinib for kinases at two different concentrations, 5 μM and 50 μM. Values represent residual activity in %.**

	**Kinase**	**Bozepinib (μM)**	
		**5**	**50**
**1**	AKT1	96	78
**2**	AKT2	100	43
**3**	AKT3	98	88
**4**	CDK/CycA	101	69
**5**	CDK/CycB1	117	74
**6**	CDK/CycE	109	64
**7**	CDK/CycA	102	65
**8**	CDK/CycE	102	66
**9**	CDK/CycE	104	81
**10**	CDK/CycD1	97	67
**11**	CDK/CycD3	98	76
**12**	CDK/p25NCK	98	75
**13**	CDK/p35NCK	100	71
**14**	CDK6/CycD1	95	64
**15**	CDK7/CycH/MAT1	96	69
**16**	CDK8/CycC	109	72
**17**	CDK9/CycT	102	69
**18**	EGF-R wt	84	17
**19**	ERBB2	101	25
**20**	ERK2	104	50
**21**	IKK- alpha	104	51
**22**	IKK-beta	113	78
**23**	IKK-epsilon	113	71
**24**	JAK3	94	43
**25**	JNK1	45	29
**26**	JNK2	77	37
**27**	JNK3	81	43

**28**	PDK1	41	27
**29**	PKC-alpha	86	24
**30**	P38-alpha	115	57
**31**	RET	79	14
**32**	STK23	95	89
**33**	STK33	102	72
**34**	VEGF-R1	91	18
**35**	VEGF-R2	82	13
**36**	VEGF-R3	84	13

Table S4. Top 25 upregulated genes in MDA-MB 468 cells after 4 hours of Bozepinib treatment.

GENE SYMBOL	GENE DESCRIPTION	FOLD CHANGE
HMOX1	heme oxygenase (decycling) 1	15,55
CYP1A1	cytochrome P450, family 1 (A), polypeptide 1	7,62
SLC7A11	solute carrier family 7, member 11	4,41
DNAJA2	DnaJ (Hsp40) homolog, subfamily A, member 2	3,52
AGPAT9	1-acylglycerol-3-phosphate O-acyltransferase 9	3,19
SCD	stearoyl-CoA desaturase (delta-9-desaturase)	3,18
UIMC1	ubiquitin interaction motif containing 1	3,14
UIMC1	ubiquitin interaction motif containing 1	3,14
LLPH	LLP homolog, long-term synaptic facilitation	2,98
NOL7	nucleolar protein 7, 27kDa	2,80
AKR1C2	aldo-keto reductase family 1, member C2	2,69
ZC3H15	zinc finger CCCH-type containing 15	2,65
UFM1	ubiquitin-fold modifier 1	2,60
SRXN1	sulfiredoxin 1 homolog (S. cerevisiae)	2,53
GCLC	glutamate-cysteine ligase, catalytic subunit	2,47
AKR1C3	aldo-keto reductase family 1, member C3 (3-alpha hydroxysteroid dehydrogenase, type II)	2,44
DDIT3	DNA-damage-inducible transcript 3	2,44
GCLM	glutamate-cysteine ligase, modifier subunit	2,38
AKR1B10	aldo-keto reductase family 1 (B10)	2,37
CTH	cystathionase (cystathionine gamma-lyase)	2,29
CYP4F11	cytochrome P450, family 4 (F), polypeptide 11	2,26
LOC644714	hypothetical protein LOC644714	2,24
ANKRD32	ankyrin repeat domain 32	2,20
FAM115A	family with sequence similarity 115, member A	2,18
TXNRD1	thioredoxin reductase 1	2,16

Table S5. Top 25 upregulated genes in MDA-MB 468 cells after 16 hours of Bozepinib treatment.

GENE SYMBOL	GENE DESCRIPTION	FOLD CHANGE
AKR1C2	aldo-keto reductase family 1, member C2	4,12
CXCL10	chemokine (C-X-C motif) ligand 10	4,00
AKR1C3	aldo-keto reductase family 1, member C3	3,01
RNU4-1	RNA, U4 small nuclear 1	2,82
RNU5E	RNA, U5E small nuclear	2,79
RNU5A	RNA, U5A small nuclear	2,59
RNU4-2	RNA, U4 small nuclear 2	2,41
RNU2-1	RNA, U2 small nuclear 1	2,30
STC2	stanniocalcin 2	2,25
SNORD116-6	small nucleolar RNA, C/D box 116-6	2,21
ASNS	asparagine synthetase (glutamine-hydrolyzing)	2,07
CTH	cystathionase (cystathionine gamma-lyase)	2,06
CHAC1	ChaC, cation transport regulator homolog 1	2,05
DDIT4	DNA-damage-inducible transcript 4	1,94
ALDH1L2	aldehyde dehydrogenase 1 family, member L2	1,93
MUC15	mucin 15, cell surface associated	1,92
AKR1B10	aldo-keto reductase family 1, member B10	1,90
PIR	pirin (iron-binding nuclear protein)	1,87
NUPR1	nuclear protein, transcriptional regulator, 1	1,86
CCL2	chemokine (C-C motif) ligand 2	1,83
CD209	CD209 molecule	1,80
DENND2D	DENN/MADD domain containing 2D	1,79
SCARNA10	small Cajal body-specific RNA 10	1,76
CYP39A1	cytochrome P450, family 39 (A), polypeptide 1	1,75
LSM14B	LSM14B, SCD6 homolog B (S. cerevisiae)	1,73

Table S6. Top 25 genes down-regulated in MDA-MB 468 cells after 4 hours of Bozepinib treatment.

GENE SYMBOL	GENE DESCRIPTION	FOLD CHANGE
LOC100131541	hypothetical LOC100131541	2,12
S100A7A	S100 calcium binding protein A7A	1,85
EFCAB4B	EF-hand calcium binding domain 4B	1,82
TNS4	tensin 4	1,79
GJA5	gap junction protein, alpha 5, 40kDa	1,66
ENC1	ectodermal-neural cortex 1 (with BTB-like domain)	1,66
MAML2	mastermind-like 2 (Drosophila)	1,64
GRAMD2	GRAM domain containing 2	1,64
CLDN1	claudin 1	1,63
DLX3	distal-less homeobox 3	1,63
AMOT	angiomotin	1,63
LCN1	lipocalin 1 (tear prealbumin)	1,62
IKZF2	IKAROS family zinc finger 2 (Helios)	1,60
APBB3	amyloid beta (A4) precursor protein-binding, family B	1,59
LOC100134868	hypothetical LOC100134868	1,58
PTCHD1	patched domain containing 1	1,58
TAS2R14	taste receptor, type 2, member 14	1,58
CX3CR1	chemokine (C-X3-C motif) receptor 1	1,57
SLC10A5	solute carrier family 10 (sodium/bile acid cotransporter family)	1,57
CITED4	Cbp/p300-interacting transactivator, with Glu/Asp-rich carboxy-terminal domain,	1,57
IRX3	iroquois homeobox 3	1,57
P2RY2	purinergic receptor P2Y, G-protein coupled, 2	1,57
FMO6P	flavin containing monooxygenase 6 pseudogene	1,56
ATP6V0A4	ATPase, H+ transporting, lysosomal V0 subunit a4	1,55
PPAP2B	phosphatidic acid phosphatase type 2B	1,55

Table S7. Top 25 genes down-regulated in MDA-MB 468 cells after 16 hours of Bozepinib treatment.

GENE SYMBOL	GENE DESCRIPTION	FOLD CHANGE
OCR1	ovarian cancer-related protein 1	4,84
VTRNA1-1	vault RNA 1-1	3,57
OLFML3	olfactomedin-like 3	3,40
LOC100131541	hypothetical LOC100131541	2,93
ATP6V0D2	ATPase, H+ transporting, lysosomal 38kDa	2,88
TNC	tenascin C	2,88
SLCO2A1	solute carrier organic anion transporter family	2,87
KRT6A	keratin 6A	2,85
CPA4	carboxypeptidase A4	2,71
GPR21	G protein-coupled receptor 21	2,67
KRT6B	keratin 6B	2,64
IGFBP5	insulin-like growth factor binding protein 5	2,63
LOC51152	melanoma antigen	2,54
TNS4	tensin 4	2,51
C9orf131	chromosome 9 open reading frame 131	2,48
VIM	vimentin	2,47
SYNE2	spectrin repeat containing, nuclear envelope 2	2,44
ANKRD36B	ankyrin repeat domain 36B	2,42
LOC100130428	IGYY565	2,39
CRISP3	cysteine-rich secretory protein 3	2,38
DKK1	dickkopf homolog 1 (Xenopus laevis)	2,29
BRIP1	BRCA1 interacting protein C-terminal helicase 1	2,27
LOC100132099	FRSS1829	2,27
HIST1H2BM	histone cluster 1, H2bm	2,25
ANKRD36B	ankyrin repeat domain 36B	2,24
